# A Systems Approach towards an Intelligent and Self-Controlling Platform for Integrated Continuous Reaction Sequences**

**DOI:** 10.1002/anie.201409356

**Published:** 2014-11-05

**Authors:** Richard J Ingham, Claudio Battilocchio, Daniel E Fitzpatrick, Eric Sliwinski, Joel M Hawkins, Steven V Ley

**Affiliations:** Department of Chemistry, University of CambridgeLensfield Road, Cambridge, CB2 1EW (UK); Pfizer Worldwide Research and DevelopmentEastern Point Road, Groton, CT 06340 (USA)

**Keywords:** continuous processing, flow reactors, integrated systems, synthetic methods

## Abstract

Performing reactions in flow can offer major advantages over batch methods. However, laboratory flow chemistry processes are currently often limited to single steps or short sequences due to the complexity involved with operating a multi-step process. Using new modular components for downstream processing, coupled with control technologies, more advanced multi-step flow sequences can be realized. These tools are applied to the synthesis of 2-aminoadamantane-2-carboxylic acid. A system comprising three chemistry steps and three workup steps was developed, having sufficient autonomy and self-regulation to be managed by a single operator.

During traditional batch-mode synthesis, researchers usually treat each step in isolation. Products are worked up, purified and analyzed prior to considering the next step in a sequence. By contrast, in the area of machine-assisted flow synthesis,[1] the ability to insert analogous in-line processing operations between the individual steps is essential for procedures consisting of more than one chemical transformation.[2–6] This requirement inspires a second design layer when planning multi-step flow procedures, which encompasses the engineering requirements for the system. This layer includes the new flexible, low cost in-line processing tools,[7–11] which enable some of the concepts of multi-step manufacturing processes to be applied within the research laboratory.[2,3,7]

Increasingly elaborate reactor assemblies rapidly become difficult to manage, and so an additional design layer must be invoked, incorporating the necessary data management.[4c] The key role of the information layer is to take overall control of the system, coordinating each module or instrument to run a prescribed sequence of events.[3] Further levels of functionality can be added to augment this base platform, to create an intelligent and responsive system.[12]

We can view any synthetic procedure in terms of these three conceptual layers (Figure 1): the first level concerns the chemical transformations that are involved in the overall sequence; the second (engineering) layer deals with the practical aspects of transferring material between the stages, to achieve a level of automation where intermediates are passed directly between the machines; and the third (information) level involves the transmission and monitoring of data, ultimately to create a self-controlling system.

**Figure 1:**
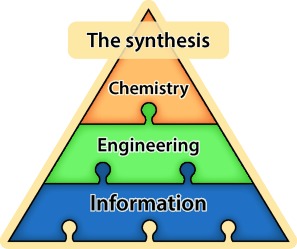
Three layers that must be considered when creating a telescoped flow synthesis procedure: transformation steps (chemical), downstream processing (engineering), and the control system (information).

Flow chemistry systems are frequently described as time-savers, because work can be delegated to electronic devices.[1] However, in our experience many chemists paradoxically spend more time at the bench when working with flow reactors than with batch equipment. A computer can help to mitigate this, by managing the different components and making programmed decisions such that the chemist need only set up and oversee the process. Thus, the availability of intelligent control systems and remote monitoring tools helps to build trust in the equipment, leading to greater working flexibility and more freedom for intellectual productivity.

Previously, we had thoroughly explored the chemistry for the flow synthesis of 2-aminoadamantane-2-carboxylic acid. With this knowledge, we could identify the challenges for creating an integrated process: the first-generation flow procedure[13] (Scheme 1) was mostly conducted in single-step operations with intermediate manual work-up operations. The integration of these steps into a single telescoped process presented a significant and illustrative challenge for the three-layer approach described herein (Figure 2, Scheme 2).

**Figure 2:**
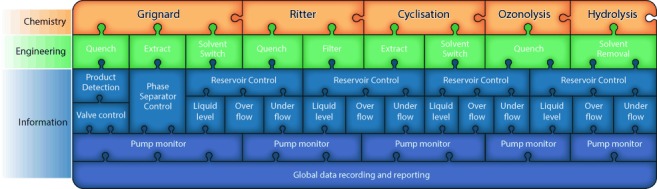
Components of the synthesis system. The chemical transformation operations are connected fluidically; a number of in-line processing operations may be associated with each. Control components such as intermediate reservoirs may interact with multiple chemistry or engineering components. Global monitoring is important for data collection and record keeping.

**Scheme 1:**
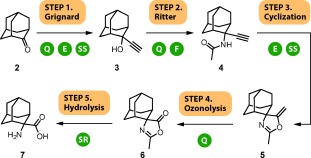
First-generation continuous flow process, with five synthetic operations. Manual operations are indicated by circular icons: Q quenching, E extraction, SS solvent switch, F filtration, SR solvent removal. Conditions: Step 1: ethynyl magnesium bromide (1), THF, 40 °C, 40 min, 90 %; Step 2: 1:2:1 H_2_SO_4_/AcOH/Ac_2_O, MeCN/AcOH, 30 °C, 7 min, 91 %; Step 3: KOH, 40:1 EtOH/H_2_O, 120 °C, 50 min, 91 %; Step 4: O_3_, CH_2_Cl_2_, 25 °C, 10 s, then solid-supported thiourea, 95 %; Step 5: HCl/AcOH/H_2_O, 150 °C, 18 min, 94 %.

**Scheme 2:**
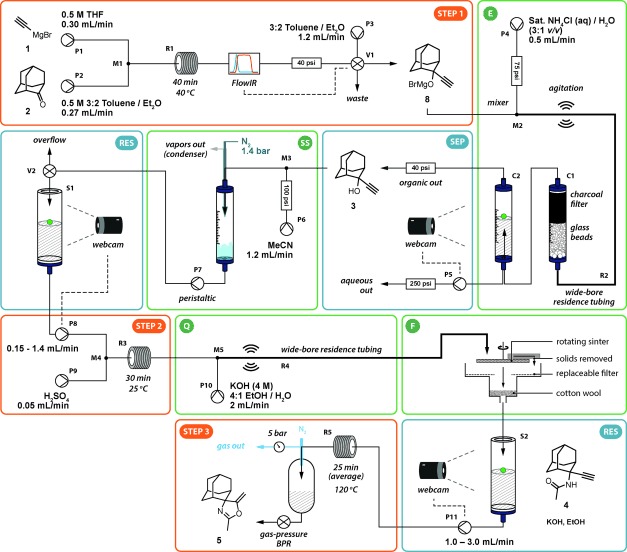
Seven-operation integrated synthesis platform. (P pump, V valve, M mixer, R reactor, C column, S reservoir). The output from the initial Grignard step is subjected to in-line quenching and then computer-controlled liquid–liquid phase separation. This solution undergoes a solvent switch and the output is stored in a reservoir before being used for the Ritter reaction stage. The acidic output is quenched with base and the resulting salts removed by a continuous filter. The filtrate is stored in a second reservoir before finally being heated to undergo cyclization.

Many of the work-up and material processing techniques that we are accustomed to in the batch mode research environment are non-trivial for machines. Fortunately, a number of tools have been developed to address these issues. For example, chemists have developed packed-tube heterogeneous catalysts and reagents to reduce the requirement for purification,[6] liquid–liquid separators to enable solvent extraction procedures,[8] continuous filtration devices[4a] and continuous chromatography for purification.[9] Continuous solvent evaporators and distillation devices can perform in-line concentration or removal of incompatible solvents.[10] We turned our attention to the application of these tools to the synthesis of compound **7**.

The first challenge involved the introduction of an aqueous quenching stream into the organic efflux following the organometallic Grignard addition (step 1). This operation had a high probability of blockage by precipitate formation, which would interrupt the process. We adopted a solution consisting of a tube-in-tube mixer (**M2**; this component injects the organic stream within and in the same direction as the quenching aqueous stream: see the Supporting Information for full details) equipped with a vibrating micromotor for mechanical agitation. The precipitation event resulting from the combination of the two streams was then managed, preventing blockages. The majority of the solids formed were re-dissolved within the residence tubing; any residue was removed by a charcoal filter (**C1**). This filter also served to give a good final contact between the phases in addition to the circulatory mixing within the immiscible slugs.[8c]

Following the quenching operation, a phase separation process was required to remove the aqueous stream. Of the many liquid–liquid phase separation technologies available,[8] we adopted a robust gravity-separation solution[8b] (see the Supporting Information) that we have observed to be minimally affected by pressure fluctuations or precipitate formation. Computer control enabled stable and autonomous operation of this device: the height of the phase boundary within the separator (**C2**) was measured using computer vision[14] and this data output was coupled to the flow rate of the pump (**P5**) that removed the lower aqueous phase.

The data output from an in-line IR spectrometer, used to detect the product **8**, was coupled to a switching valve (**V1**) and to the downstream pumps, to ensure that the aqueous extraction and the following steps did not start until **8** was delivered from the first stage. This was an important consideration to prevent solvent and energy wastage, and was one of the advantages provided by the integrated control layer.

The next challenge also concerned the first reaction stage. Commercial availability dictated that the Grignard reagent **1** was used in a solution of THF, and so this solvent had to be removed before the Ritter reaction stage to prevent polymerization under the acidic conditions. Preliminary tests using a nebulizing evaporator device[10] to perform a solvent exchange found that more than 95 % of the THF was removed when the process stream was introduced in a 1:2 ratio with an acetonitrile co-stream. The resulting solution was withdrawn from the evaporation chamber by a peristaltic pump (**P7**).

The peristaltic pump produced a low-pressure stream with a pulsing flow rate. Therefore, a reagent reservoir[3] (**S1**; see the Supporting Information for schematic and operation details) was introduced to buffer this variation in flow and to enable an HPLC pump (**P8**) fed by this reservoir to generate a pressurized reagent stream for the subsequent Ritter stage.[15] Furthermore, its flow rate could be adjusted based on the amount of available material. To achieve this, the liquid level was measured by computer-vision monitoring of a colored float,[3] using the same camera as that used in the previous extraction step. A control algorithm was integrated into the information layer to match the flow rate of reagent injection into the Ritter step to that of the output from the evaporator (Figure 3). The algorithm would also slow and stop the pump if a problem occurred in a previous stage. In the event of a problem within the Ritter step causing pump **P8** to stop, output from the evaporator was redirected (valve **V2**) to prevent overflow of the reservoir. Importantly, the reservoir allowed the stages to be fluidically separate, so that one step could be stopped for maintenance without necessarily affecting the others. Similarly, the software algorithms for each stage were compartmentalized to create a simple and flexible system, whilst still allowing all the devices to be automatically shut down in the event of an unrecoverable failure in any one of them.

**Figure 3:**
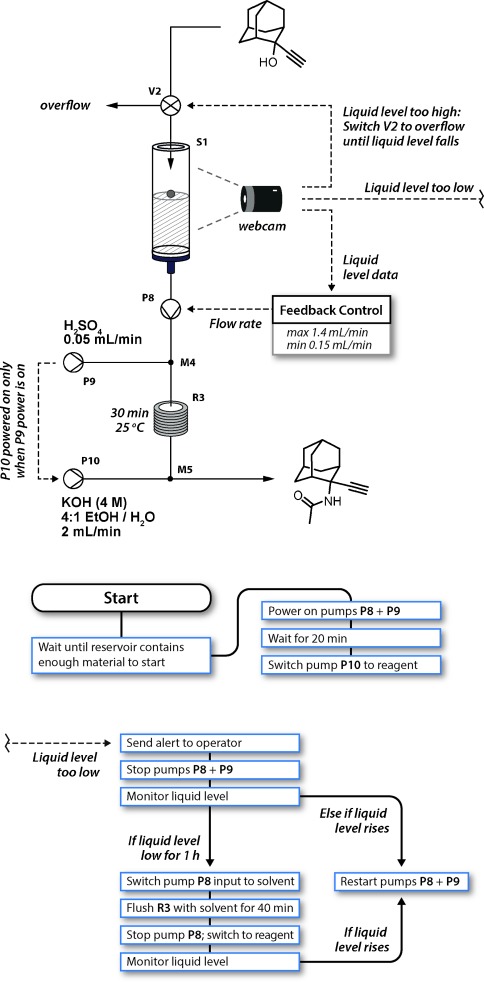
Control algorithms for the components (top) of the Ritter reaction (step 2 in Schemes 1 and 2). The main control sequence (middle) started the Ritter step as soon as sufficient material had been collected from the first step. Whilst running, a simple feedback control algorithm equalized the flow rates between the two steps, slowing or stopping pump P8 if the liquid level dropped. A washing sequence (bottom) was triggered if the reagent pump was stopped for over an hour. Finally, the output was redirected to an overflow if the liquid level rose too high, to prevent flooding.

The filtrate, containing compound **4**, was stored in a second monitored reservoir (**S2**) and then delivered by an HPLC pump (**P11**) to a heated coil reactor (**R5**) to perform the 5-(enol)*exo*-dig cyclisation. Blockages from crystallization of the product were encountered when a spring-based BPR was used; an alternative gas-BPR system[11] provided pressure effectively without blockage even in the presence of small quantities of solids.

At this point in the sequence, the base had to be removed to prevent salt precipitation during the ozonolysis stage, and to simplify the purification of the final product. Therefore, the synthesis sequence was broken and manual solvent evaporation and aqueous work-up was performed.

The remaining two chemistry steps were achieved using a simple two-step flow procedure with only minor modifications to the first-generation conditions (Scheme 3). In a less toxic acetone/water solvent system, the carbonyl oxide intermediate was decomposed in situ,[16] avoiding the requirement for the stoichiometric solid-supported thiourea reagent involved in the first-generation synthesis. The hydrogen peroxide by-product of the reaction with water was decomposed into H_2_O and O_2_ using a packed bed of amorphous MnO_2_ catalyst[17] (**R7**). The output of this reaction was consistent with a 1:1 mixture of compounds **6** and **9**. Pleasingly, when this mixture was subjected to the hydrolytic conditions, complete conversion to the desired product **7** was observed.

**Scheme 3:**
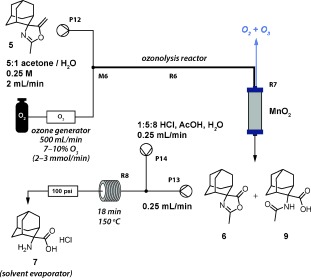
Final chemistry steps: ozonolysis and hydrolysis.

The hot output from the reactor (**R8**) was directed into a second nebulizing evaporator. All of the volatile solvents and by-products were removed, effectively spray-drying the product **7** onto the walls of the evaporation chamber and allowing for a simple collection procedure to complete the synthesis.

The seven-operation reactor (steps 1–3) was operated for periods of up to six hours in each day (allowing time to check the reactor beforehand and perform a thorough purge afterwards), providing an output of 8 mmol h^−1^ of pure material as detected by ^1^H NMR analysis. Further work is needed to reach our goal of running for more than 48 h; primarily this requires advances in pumping technology, as the main challenge in this case was blockages created by slurries. However, the reservoir check-points enabled us to run the system for an extended period, as an individual step could be isolated for intervention by the operator without significantly affecting the others. Additional software logic to perform a safe shut-down in the event of a blockage could allow unattended operation, but in general the operator is an important part of the system, and this means that the mean failure time of any one component can be relatively low without compromising the entire system. Importantly, the use of commodity hardware allows for significant flexibility: for example, we anticipate that this system (hardware and software) could be constructed in a single day assuming each of the work-up components was available as a pre-built unit. Furthermore, small changes could be implemented very quickly and this enabled us to experiment with different reactor configurations during the development process.

In conclusion, flow chemistry has many benefits, such as offering better safety, reliability, and sustainability, as well as access to novel pathways using intensive conditions; but to make effective multi-step procedures, in-line processing tools must be used (the engineering layer of the system).[18] To control the resulting complex reactor configurations, an information layer must also be added. This combination allows an entire process to be operated as an integrated system, whilst maintaining the identity of the individual components, which provides expandability as well as the flexibility that is required in a research laboratory. Decoupling the algorithms from the equipment in this way will enable smoother transitions from discovery to manufacturing, to allow novel reactivity and techniques to rapidly deliver impact on synthesis programs.
