# Supplementary material for: A Systems Approach towards an Intelligent and Self-Controlling Platform for Integrated Continuous Reaction Sequences
Source: Angew Chem Int Ed Engl. 2014 Nov 5;54(1):144–8. doi: 10.1002/anie.201409356 (PMC4502965; doi:10.1002/anie.201409356)
Supplement: Supplementary file 1 [file anie0054-0144-sd1.pdf]

Angewandte Chemie
Eine Zeitschrift der Gesellschaft Deutscher Chemiker

Supporting Information

© Wiley-VCH 2014

69451 Weinheim, Germany

A Systems Approach towards an Intelligent and Self-Controlling Platform for Integrated Continuous Reaction Sequences**

*Richard J. Ingham, Claudio Battilocchio, Daniel E. Fitzpatrick, Eric Sliwinski, Joel M. Hawkins, and Steven V. Ley**

anie_201409356_sm_miscellaneous_information.pdf

Contents

General experimental information 3
Flow reactor configuration 3
Tube-in-tube tee mixer 6
Liquid-liquid extractor 7
Liquid reservoir 8
Continuous filtration device 9
Nebulising evaporator 9
Gas-BPR system 9
Software control 10
Computer vision 11
References 12

3D Models

Interactive 3D models of the devices described herein are available on the WWW under:

http://www.leygroup.ch.cam.ac.uk/suppl/anie.2014

General experimental information

HPLC grade acetonitrile and concentrated sulfuric acid were purchased from Fisher Scientific, and absolute ethanol from Sigma-Aldrich, and were used without purification; THF, Diethyl Ether, and Toluene are purchased from Fisher Scientific and distilled before use.

2-Adamantanone was purchased from either Sigma-Aldrich or Alfa Aesar and used without purification.

Ethynyl magnesium bromide was obtained from Sigma-Aldrich (product number 346152, SureSeal™ bottle) and used as purchased.

Activated charcoal powder was obtained from Breckland Scientific Supplies Ltd.

Unless specified otherwise, all connective tubings were 1 mm I.D. PFA.

Flow reactor configuration

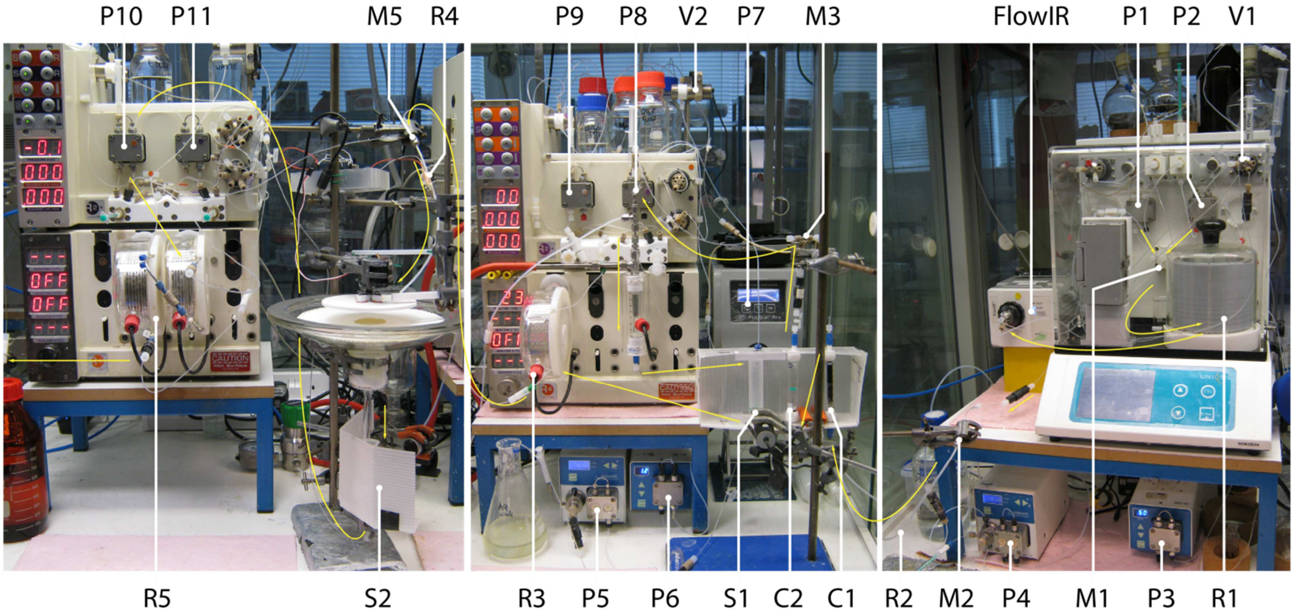

The yellow arrows indicate the approximate flow path between devices.

Reagent Stream A

A 0.5 M solution of ethynyl magnesium bromide (**1**), in a SureSeal™ bottle equipped with an inert gas inlet connected to a nitrogen manifold.

Reagent Stream B

A 0.5 M solution of 2-adamantanone (**2**) in Toluene/Et_2O (3:2, *v/v*), stored in a sealed flask under nitrogen (manifold).

Stage 1

Reagent Stream A was delivered at a flow rate of 0.30 mL min^{-1} (**P1**; Uniqsis FlowSyn) and combined at tee mixer **M1** (Uniqsis FlowSyn integrated tee mixer) with Reagent Stream B delivered at a flow

rate of 0.27 mL min^{-1} (**P2**; Uniqsis FlowSyn) and then heated to 40 °C in reactor **R1** (Ø 1 mm, PFA, 14 mL: 37 minutes residence time). A 100 psi back pressure regulator was employed to regulate the pressure of the system. After exiting the coil the flow stream passed through a Mettler-Toledo FlowIR™ spectrometer in order to detect the presence of the Grignard product **8**. The detector was linked to valve **V1** (Uniqsis FlowSyn integrated loop-switching valve) by software control (see page 10) and the output stream was directed to the next stage only when this product was detected.

Quenching Stream C

A saturated solution of NH_4Cl, combined in a ratio of 3 : 1 with distilled water (250 mL).

Stage 2

The reaction solution was combined with Quenching Stream C, delivered at a flow rate of 0.5 mL min^{-1}, (**P4**, Knauer Smartline S100) using a tube-in-tube mixer (**M2**, see page 6); the output was directed into reactor **R2** (Ø 2.4 mm, 600 mm length, ETFE). A micro vibration motor was attached to **R2**, reducing the occurrence of blockages at **M2**. The plug-flow system formed was delivered to glass column reactor **C1** (Ø 6.6 mm, 100 mm length, glass) packed with charcoal and glass beads (diameter 0.5 – 0.7 mm; a layer of 4 mm diameter glass beads kept the smaller beads in place) in order to remove magnesium aggregates by filtration. The resulting clear biphasic system was then delivered to liquid/liquid separating column **C2** (Ø 10 mm, length 100 mm, glass). The aqueous phase was withdrawn continuously by pump **P5** (Knauer Smartline S100) at a flow rate based on the position of the interface (see page 7). The organic layer was forced out of the top of the column into the next stage. A pressure differential created by the two back-pressure regulators prevented undesired siphoning of liquid through pump **P5**.

Stage 3

The organic output from **C2** was combined at tee mixer **M3** (Ø 0.5 mm, PEEK) with a stream of MeCN delivered at 1.2 mL min^{-1} by pump **P6** (Knauer WellChrom K120). The mixture was introduced into a nebulising evaporation device which was heated to 22 °C (see page 9) along with an N_2 stream with pressure 1.4±0.1 bar. The remaining solution was withdrawn from the bottom of the evaporation chamber using a peristaltic pump (**P7**) at a flow rate of 1 mL min^{-1}. The solution was collected in reservoir **S1**. The volume of material within **S1** was measured by a digital camera (see page 11) and this data was used to control the flow rate of pump **P8** (Vapourtec R2+) in order to match the input and output flow rates.

Stage 4

This solution was pumped at a flow rate of 0.15 – 1.4 mL min^{-1} by pump **P8** and combined at mixer **M4** (Ø 0.5 mm, ETFE) with a stream of concentrated sulfuric acid which was delivered at 0.05 mL min^{-1} by pump **P9** (Vapourtec R2+ with acid-resistant fittings). The mixture was passed through reactor **R3** (Ø 1 mm, PFA, 10 mL: minimum 7 minutes residence time).

Quenching Stream D

A 4 M solution of KOH in EtOH/H_2O (4:1, *v*/*v*).

Stage 5

The output of reactor **R3** was combined with Quenching Stream D delivered by pump **P10** (Vapourtec R2+) in a tube-in-tube mixer (**M5**, see page 6); the output was directed into reactor **R4** (Ø

2.4 mm, 300 mm length, ETFE). Another vibrating device was attached to **R4**, reducing the occurrence of blockages at **M5**. The stream exiting this reactor was directed onto the continuous filtration device (see page 9) which removed the salts formed by the quenching of the acid to leave a basic EtOH solution of compound **4**, which was collected in reservoir **S2**.

Stage 6

The solution collected in reservoir **S2** was pumped (**P11**) to reactor **R5** (Ø 2.5 mm, stainless steel, 50 mL: minimum 25 minutes residence time) heated to 120 °C. Following the reactor was a gas-BPR system (see page 9) pressurised at 5 bar using the local N_2 gas supply. The reactor output was released from the holding vessel periodically using the manual valve, and collected for manual solvent evaporation and aqueous extraction.

Stage 7

A solution of 2-methyl-4-(adamantane-2′-spiro)-5-methylidene oxazoline (**5**) in acetone/H_2O (5:1, *v/v*, 0.25 M) was delivered at 2.00 mL min^{-1} by pump **P12** (Knauer WellChrom K120) to meet a stream of ozone **G1** (0.5 bar, flow rate 500 mL min^{-1}) and these were combined in a Y-mixer (Ø 0.5 mm, PEEK) and reacted directly through a 1.4 mL PFA tube reactor **R8** (Ø 2.5 mm, 180 mm length, PFA, 20 s residence time). The solution was directed into column **C4** (Ø 10 mm, length 100 mm, glass) containing amorphous MnO_2 (2 g) through which the liquids percolated and the gases could escape. The resulting solution was collected and then pumped at 0.25 mL min^{-1} by pump **P11** (Vapourtec R2+) to be combined at a tee mixer (Ø 0.5 mm, ETFE) with a stream of HCl/AcOH/H_2O (1:5:8, *v/v/v*) delivered at 0.25 mL min^{-1} by pump **P12** (Vapourtec R2+) and directed into reactor **R9** (Ø 2.4 mm, 10 mL, PFA) heated to 150 °C. The output of the reactor was finally directed into an unheated solvent evaporator device (see page 9). The solid recovered from this stage was washed with hot acetonitrile (2 × 50 mL per gram of product) and diethyl ether (2 × 50 mL per gram of product), before being recrystallized from MeOH (0 °C) to give the pure product (**7**).

Tube-in-tube tee mixer

Configuration A (Swagelok reducers):

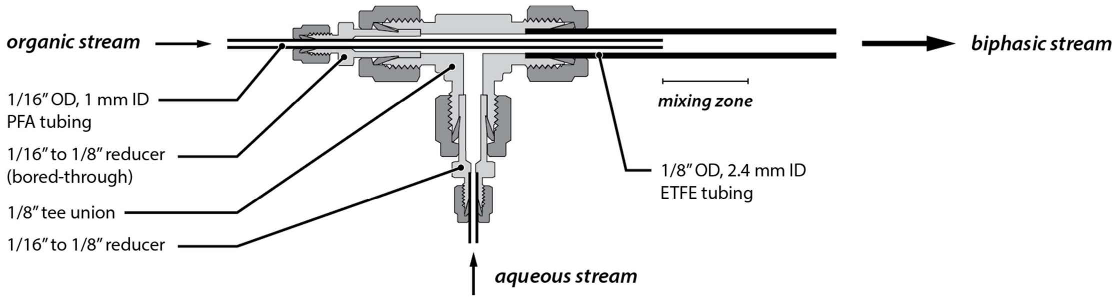

Parts: Swagelok 1/8“ tee union (SS-200-3)
Swagelok 1/16“ to 1/8“ reducers (SS-100-R-2 and SS-100-R-2-BT)
1/8“ x 2.4 mm ETFE tubing (Kinesis P/N 1530)

Configuration B (PTFE tubing reducers):

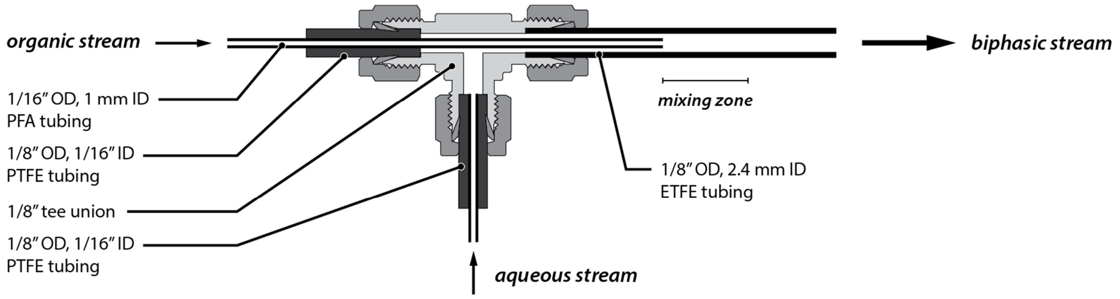

Parts: 1/8“ x 1.5 mm PTFE tubing (Kinesis P/N 008T32-150-10)

Due to the lower pressure the use of a 1/8“ tubing sheath was sufficient to maintain the seal around the 1/16“ tubing in the 1/8“ port.

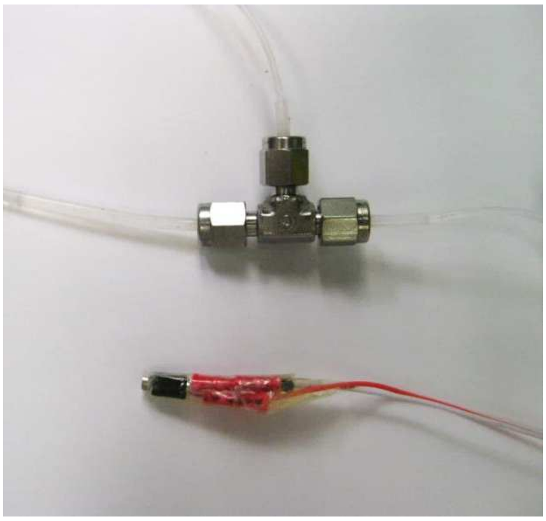

A 3 V micro DC vibration motor was tightly attached (using e.g. clear sticky tape) to the large-bore tubing to prevent solid aggregation within the mixer or tubing. A small piece of putty adhesive (e.g. Blu-Tack) was placed between the motor and the tubing to prevent mechanical wearing of the tubing.

Liquid-liquid extractor

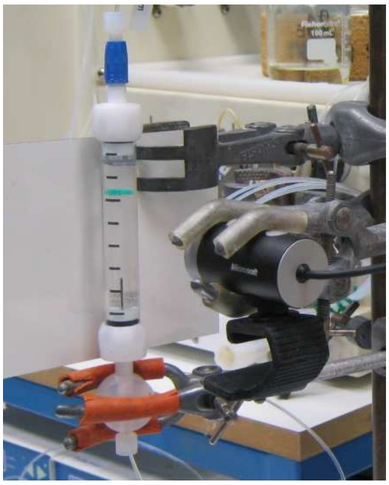

Detail of lower fitting:

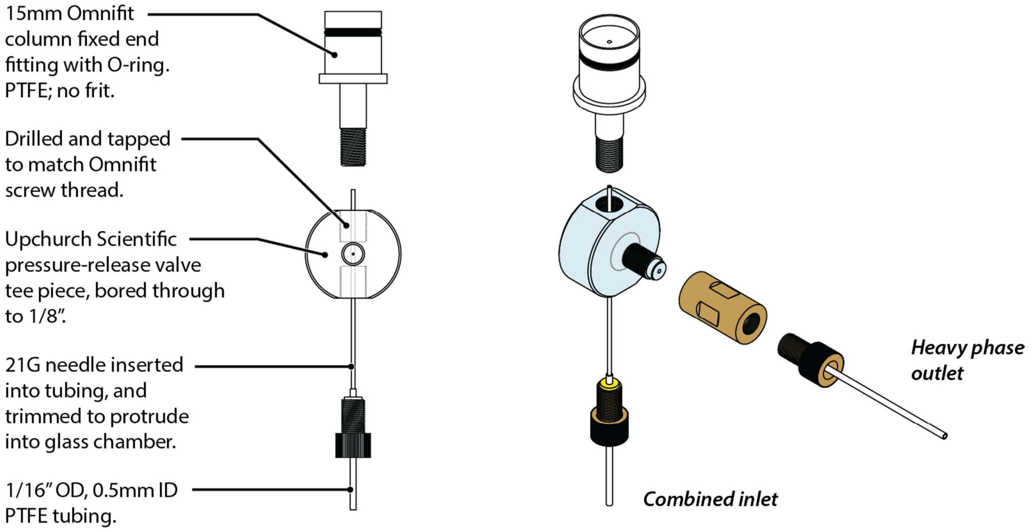

Parts: Omnifit SolventPlus Glass Column 10 mm x 100 mm, 2x fixed ends. (Kinesis P/N 006SCC-10-10-FF).
Upchurch Pressure release valve tee for 1/16" OD, ¼-28 fittings. (IDEX P/N P-612). This is re-tapped to accommodate the Omnifit column end-piece.
ETFE 1/16" OD ¼-28 SuperFlangeless fitting nuts (IDEX P/N P-254X)
ETFE 1/16" OD SuperFlangeless ferrule (IDEX P/N P-259X)
Float: Segment of polyethylene plunger from a 1 mL PP/PE disposable syringe (Sigma-Aldrich P/N Z230723).

A biphasic stream entered through the 21G needle inlet. The phases split under gravity, and the interface was marked by a PE float.

The lighter phase exited the column through the top end; the exit port was protected with a 20 µM PTFE frit (Omnifit). The heavy phase exited through the bottom port, around the needle and out of the tee connector.

Liquid reservoir

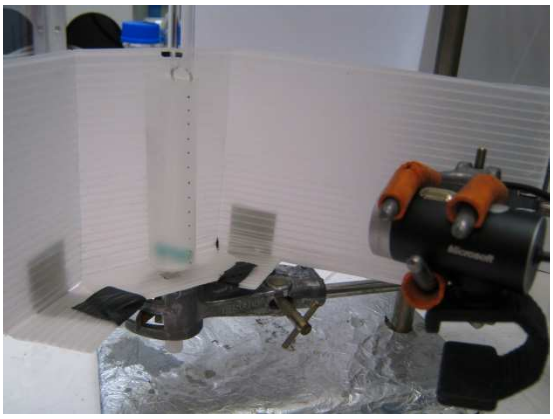

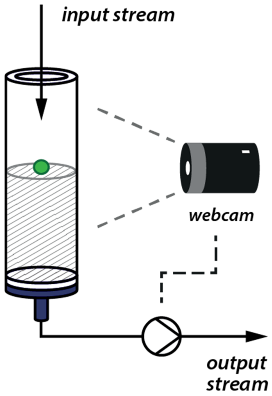

Reservoir body: 20 mL empty SPE cartridge (e.g. Agilent Bond Elut) with 20 µm Polypropylene Frit.
Reservoir float: Manufactured from a 1 mL PP/PE syringe (Sigma-Aldrich P/N Z230723). See [1].
Fittings: ETFE Adapter, Female Luer to Male 1/4"-28 Flat Bottom (IDEX P/N P-624); Delrin Threaded Union (1/4"-28) Thru-Threads (IDEX P/N P-603).
Camera: Microsoft LifeCam Cinema.

A plastic surround was added to the cartridge to provide a blank background for the camera. Dots are drawn on the front of the reservoir to trigger the camera's autofocus at the correct focal plane.

The image was filtered using the SimpleCV library for Python to locate the green float and determine its height within the image (see page 11). This data was used as the input for a feedback loop to control the flow rate of the output pump to iteratively match the flow rates of the input and output.

Continuous filtration device

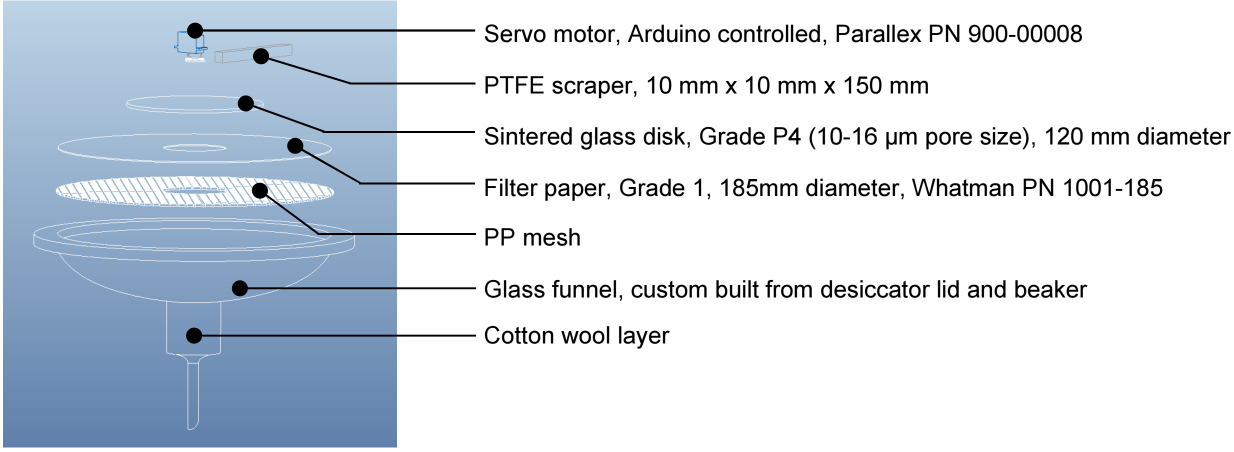

A slurry stream was allowed to drip onto a sintered glass disk attached to a servo motor whose rotation was controlled using an Arduino circuit. As this disk rotated, solids accumulated on the surface of the sinter while liquids were able to pass through. A PTFE scraper was used to remove the solid layer onto replaceable filter paper.

The bottom of the glass sinter was tapered to a point to guide liquid drops to the centre of the disk. Liquid drops fell through holes cut into the filter paper and supporting PP mesh onto a cotton wool layer. A liquid reservoir collected filtrate from the base of the glass funnel structure.

Nebulising evaporator

For details, please see Supporting Information of [2].

Gas-BPR system

For details, please see Supporting Information of [3]. N.B. This device is not shown in the reactor configuration photograph.

Software control

Connections between machines and the control computer were made using the local area network (LAN). Uniqsis, Vapourtec and Knauer machines were connected via Brainboxes ES-701 and ES-257 ethernet-to-serial adapters (http://www.brainboxes.com/), each configured with a fixed IP address on the local network. The control computer was powered by Ubuntu Linux (http://www.ubuntu.com/) and our software control platform (*Octopus*; open source: https://github.com/richardingham/octopus) was installed on this machine. Two webcams were connected to this computer (NB each must be connected to a separate USB root hub on the computer, this may require additional PCI USB adapters to be installed).

The running control system was accessed using a web browser, directed to a web server (integrated with *Octopus*) running on the control computer.

The following diagrams are schematic of the software control algorithm. The programme file (Python) can be accessed at https://gist.github.com/richardingham/4ac997b5d720a4f7384f

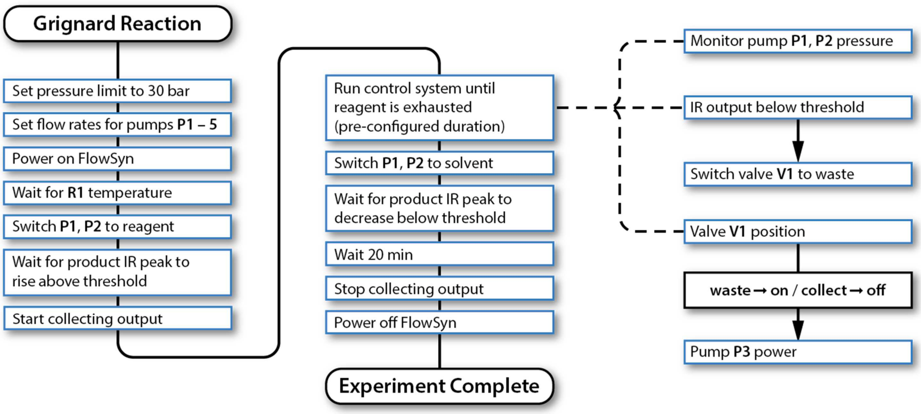

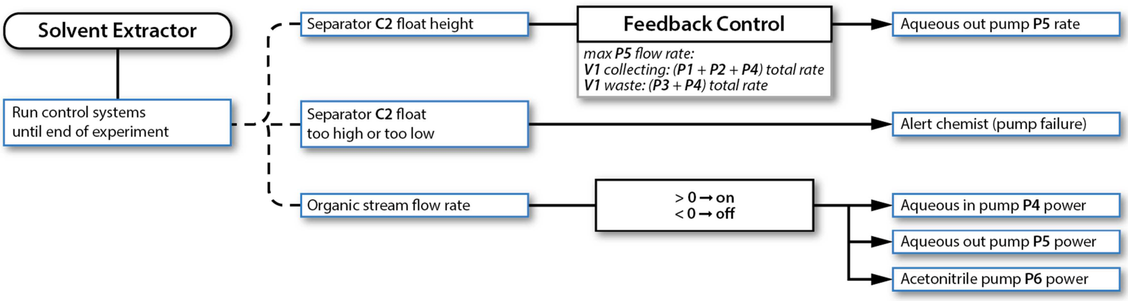

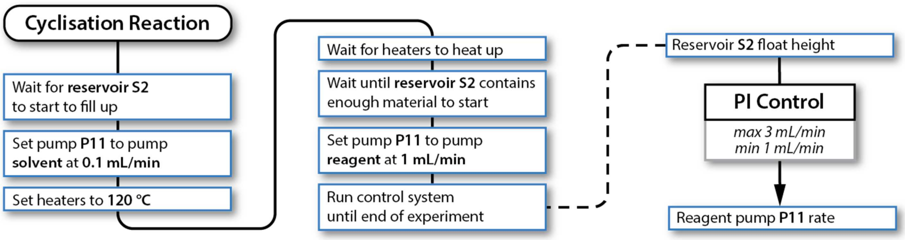

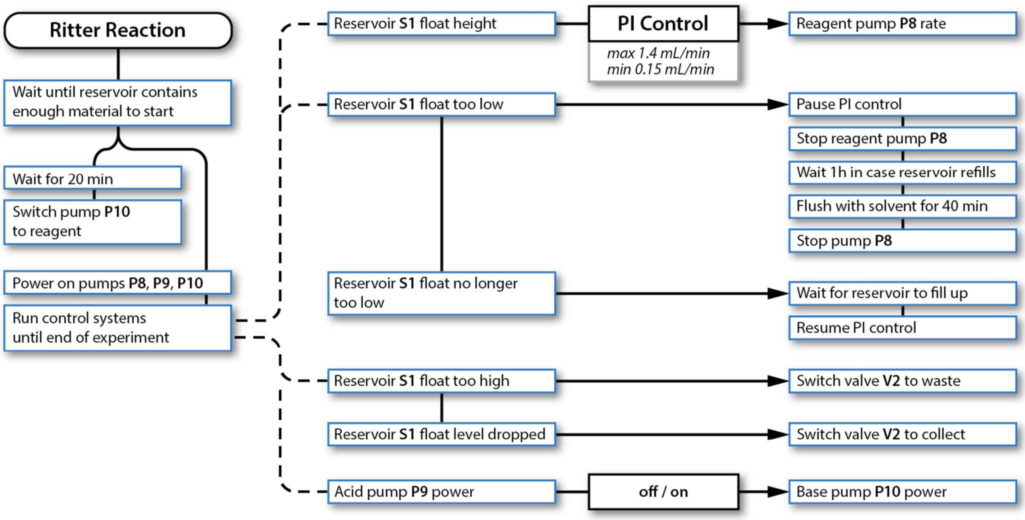

Computer vision

The computer vision was built into the *Octopus* system. See:
https://github.com/richardingham/octopus/blob/master/octopus/image/tracker.py

The correct video source was defined in the control programme for each of the two cameras. There were two computer-vision adapters, one of which looked for a single coloured object, the other for multiple objects. The colour to search for was pre-programmed in the control protocol (and was green by default, to match the PP/PE syringe plungers commonly used in our laboratory) and thus a plain background for the image is recommended. In our case, white plastic surrounds were cut and positioned behind the glass columns of interest. In the case of multiple object observation, the system used the first frame recorded to determine the horizontal location of the coloured objects and from then on looked for objects within a horizontal window of the original positions. The vertical position of the object in the image frame was returned to the control programme, which was then used as an input variable in e.g. a feedback loop.

References

[1] R. J. Ingham, *figshare* **2013**, DOI 10.6084/m9.figshare.881895.

[2] B. J. Deadman, C. Battilocchio, E. Sliwinski, S. V. Ley, *Green Chem.* **2013**, *15*, 2050–2055.

[3] C. Battilocchio, B. J. Deadman, N. Nikbin, M. O. Kitching, I. R. Baxendale, S. V Ley, *Chem. Eur. J.* **2013**, *19*, 7917–7930.
